# Sicilian Litchi Fruit Extracts Induce Autophagy versus Apoptosis Switch in Human Colon Cancer Cells

**DOI:** 10.3390/nu10101490

**Published:** 2018-10-12

**Authors:** Sonia Emanuele, Antonietta Notaro, Antonio Palumbo Piccionello, Antonella Maggio, Marianna Lauricella, Antonella D’Anneo, Cesare Cernigliaro, Giuseppe Calvaruso, Michela Giuliano

**Affiliations:** 1 Laboratory of Biochemistry, Department of Experimental Biomedicine and Clinical Neurosciences (BIONEC), University of Palermo, 90127 Palermo, Italy; sonia.emanuele@unipa.it (S.E.); marianna.lauricella@unipa.it (M.L.); cesare.cernigliaro@unipa.it (C.C.); 2 Department of Biological, Chemical and Pharmaceutical Sciences and Technologies (STEBICEF), University of Palermo, 90133 Palermo, Italy; antonietta.notaro@unipa.it (A.N.); antonio.palumbopiccionello@unipa.it (A.P.P.); antonella.maggio@unipa.it (A.M.); antonella.danneo@unipa.it (A.D.); giuseppe.calvaruso@unipa.it (G.C.)

**Keywords:** *Litchi chinensis*, anti-tumor activity, autophagy, colon cancer

## Abstract

*Litchi chinensis Sonnerat* is a tropical tree whose fruits contain significant amounts of bioactive polyphenols. Litchi cultivation has recently spread in Sicily where the climate conditions are particularly favorable for this crop. Recent findings have shown that Litchi extracts display anti-tumor and pro-apoptotic effects in vitro, but the precise underlying mechanisms have not been fully elucidated. In this study, we report for the first time the effects of Sicilian litchi fruit extracts on colon cancer cells. The results indicated that litchi exocarp, mesocarp and endocarp fractions reduce the viability and clonogenic growth of HT29 cells. These effects were due to cell cycle arrest in the G2/M phase followed by caspase-dependent cell death. Interestingly, litchi exocarp and endocarp triggered a precocious autophagic response (16–24 h), which was accompanied by an increase in the level of autophagy related 1/autophagy activating kinase 1 (ATG1/ULK1), beclin-1, microtubule associated protein 1 light chain 3 (LC3)-II and p62 proteins. Autophagy inhibition by bafilomycin A1 or beclin-1 silencing increased cell death, thus suggesting that autophagy was initially triggered as a pro-survival response. Significant effects of Litchi extracts were also observed in other colon cancer cells, including HCT116 and Caco-2 cells. On the other hand, differentiated Caco-2 cells, a model of human enterocytes, appeared to be insensitive to the extracts at the same treatment conditions. High-Performance Liquid Chromatography–Electrospray Ionization-Quadrupole-Time-Of-Flight HPLC/ESI/Q-TOF evidenced the presence of some polyphenolic compounds, specifically in exocarp and endocarp extracts, that can account for the observed biological effects. The results obtained suggest a potential therapeutic efficacy of polyphenolic compounds purified from Sicilian Litchi fractions for the treatment of colon cancer. Moreover, our findings indicate that modulation of autophagy can represent a tool to improve the effectiveness of these agents and potentiate the anti-tumor response of colon cancer cells.

## 1. Introduction

*Litchi chinensis Sonnerat* is a fruit tree belonging to the Sapindaceae family, originally cultivated in China and subsequently spread to tropical and sub-tropical areas worldwide [1,2,3]. Cultivation of the litchi tree has been recently launched in Sicily (Italy), where the climatic conditions are particularly favorable for planting and crop of tropical plants [4,5]. The fruit is known for its good taste and nutritional properties [3]. Recent studies have shown that litchi pulp (mesocarp) contains bioactive compounds, including polysaccharides with strong antioxidant activities [6,7]. In addition, Huang et al. have also provided evidence that litchi pulp displays anti-tumor and immunomodulatory effects both in vitro and in vivo [8].

Other non-edible parts of the litchi fruit are also employed in popular Chinese medicine. Litchi seeds (endocarp) are used as analgesic to relieve gastralgia, cough and neuralgia [3]. Moreover, Hsu et al. have shown that litchi seed extract exerts anti-tumor and pro-apoptotic actions in human colorectal carcinoma cells [9]. However, the precise mechanism of action for apoptosis induction remains to be elucidated. Litchi peel (exocarp) has been shown to contain active flavonoids and anthocyanins which display anti-oxidant properties and can exert anti-cancer effects [10]. The anti-tumor action of litchi exocarp was found in human breast cancer cells as well as breast cancer mouse xenografts [11].

Autophagy is a highly conserved process that consists of the degradation of cellular components and nutrients to maintain cell homeostasis and survival during stress conditions. It can result in either cell survival or cell death depending on various circumstances [12]. The autophagic process is highly regulated by autophagy related gene products, named autophagy related (ATG) proteins. In the first phase of autophagy, a central component is Unc-51 like autophagy activating kinase (ULK1), a kinase encoded by the *ATG1* gene, which triggers the recruitment of other ATG proteins, including beclin-1, a component of the class III PI-3K complex, and ATG12 and ATG5 to form the phagophore [13]. Concomitantly, a cytosolic form of the microtubule associated protein 1 light chain 3 (LC3) protein (LC3-I) forms the LC3-phosphatidylethanolamine conjugate (LC3-II), which is recruited to autophagosomal membranes and therefore acts as a process marker [14,15].

In cancer cells, autophagy plays an important role as a tumor promoter or exerting tumor suppressor functions [16,17]. Tumor cells can indeed activate a pro-survival autophagic process in starvation or hypoxic conditions and increase growth and aggressiveness [18]. On the other hand, several studies suggest that autophagy can prevent tumor initiation [19]. Moreover, the induction of autophagic cell death can represent a tool for targeting tumor cells, particularly when resistance to classic apoptosis occurs. Autophagy can thus provide a useful method to limit tumor progression and enhance the efficacy of anti-cancer treatments. However, in many cases, triggering autophagic flux may represent a defensive cell mechanism against cytotoxic treatments and thus inhibiting the process may result in potentiating cell death [20].

As it is known that climatic conditions can influence the chemical composition of the fruit, we aimed to investigate the cytotoxic effects of litchi cultivated in Sicily. In this paper, we provide evidence that different separated fractions of Sicilian Litchi fruit induce colon cancer cell death through different mechanisms. In particular, we demonstrated for the first time that Litchi exocarp and endocarp extracts induce autophagy within the first phase of treatment which was interpreted as a pro-survival response that precedes cancer cell death.

## 2. Materials and Methods

### 2.1. Reagents

Reagents were from Sigma (Milan, MI, Italy), except when otherwise reported. Bafilomycin A1 was from Alfa Aesar, (Thermo Fisher Scientific, Waltham, MA, USA). Stock solutions were prepared in dimethyl sulfoxide (DMSO) and opportunely diluted in culture medium. The final concentration of DMSO never exceeded 0.01%, the concentration that was experimentally determined to have no discernible effect. The antibodies against caspase-3, caspase-9, poly-ADP-ribose-polymerase-1 (PARP-1) and Beclin-1 were purchased from Cell Signaling (Beverly, MA, USA); against p62, γ-tubulin from Sigma (Milan, MI, Italy); and against LC3 from Novus Biologicals (Cambridge, UK).

### 2.2. Preparation of Litchi Fruit Extracts

Litchi fruits were washed and the three fractions (peel, pulp and seed) were separated. The peel and the seed were cut into small pieces and lyophilized. Afterwards, the small pieces were ground using a stainless-steel grinder. The powders obtained were used to prepare the extracts using a solution of 50% ethanol in phosphate-buffered saline (PBS). The pulp was collected, homogenized in PBS, centrifuged for 10 min at 35,000 × *g* and the supernatant obtained was filtered and lyophilized. For extraction, 75 mg of the three preparations were suspended in 1 mL of 50% PBS/ethanol and incubated at 37 °C overnight. The extracts were then centrifuged at 800 × *g* for 10 min and the supernatant obtained was further centrifuged at 15,500 × *g* for 10 min.

### 2.3. Cell Cultures

Human colon cancer HT29, HCT116 and Caco-2 cells were purchased from American Type Culture Collection, Rockville, MD, USA. Human colon cancer HT29 and HCT116 cell lines were cultured in Roswell Park Memorial Institute (RPMI) 1640 medium supplemented with 10% fetal bovine serum (FBS), 1 mM glutamine, non-essential amino acids and antibiotic antimycotic solution, as previously reported [21].

To induce cell differentiation, Caco-2 cells were cultured according to the protocol proposed by Natoli et al. [22].

### 2.4. Evaluation of Polyphenol Contents

The final crude extracts were used to determine the amount of polyphenols using the colorimetric Folin-Ciocalteu method as described by Cicco et al. [23], employing gallic acid as a calibration standard. The content of total phenols was reported as mg of gallic acid equivalent (GAE)/g dry mass total extracts.

### 2.5. Evaluation of Cell Viability

The effect of the compounds on cell viability was determined by a 3-(4,5-dimethyl-thiazol-2-yl)-2,5-diphenyltetrazolium bromide (MTT) assay as previously described [24]. Values reported in the results are expressed as the percentage of control cell viability and are the means ± standard deviation (SD) of four independent experiments.

### 2.6. Clonogenic Assay

Clonogenic assays are the method of choice to detect cells that have retained the capability for producing a large number of progeny. Following the protocol previously reported [25], HT29 cells were plated at a density of 300 cells/well in 6-well tissue culture plates. After 48 h, before the cells started to replicate, the cultures were treated with litchi extracts for 8 days and the medium was replaced every 4 days. Then, cultures were stained with 0.1% w/v Coomassie blue (500 µL/well), washed and the number of colonies was quantified using a light microscope.

### 2.7. Flow Cytometry Analyses

Cell death was assessed by staining with propidium iodide (PI), a membrane impermeable dye which is generally excluded from viable cells. Cells were harvested, washed with phosphate-buffered saline (PBS) and incubated for 10 min at 4 °C in a solution of PBS containing 2 μg/mL PI. After staining, red fluorescence was measured using the FL3 channel using a 620-nm band pass (BP) filter by a Coulter Epics XL flow cytometer (Beckman Coulter, Brea, CA, USA). Data reported in the results are the means ± SD of three independent experiments.

To analyze cell cycle distribution, cells were resuspended in a hypotonic solution containing 25 μg/mL propidium iodide, 0.1% sodium citrate, 0.01% Nonidet P-40 and 10 μg/mL RNase A [26]. The cell cycle phase distribution was evaluated by a Coulter Epics XL flow cytometer (Beckman Coulter) using Expo32 software. The percentage of cells in the subG0/G1 phase was considered as an index of DNA fragmentation. The results shown are representative of three independent experiments.

### 2.8. Monodansylcadaverine (MDC) Labeling

MDC staining of autophagic vacuoles was performed to detect the autophagic flux, as previously described [15]. After treatments, cells were washed with PBS, stained with 0.05 mM MDC and incubated at 37 °C for 10 min. Then, cells were washed three times with PBS and immediately analyzed under a Leica DMR fluorescence microscope (Wetzlar, Germany) using an appropriate 4′,6-diamidino-2-phenylindole (DAPI) filter. Images were photographed and captured by a computer-imaging system (Leica DC300F camera and Adobe Photoshop for image analysis).

### 2.9. Western Blotting Analysis

Protein extracts were prepared by washing the cells in PBS and incubating for 20 min in ice-cold lysis buffer supplemented with a protease inhibitor cocktail, as previously reported [24], followed by sonication. Proteins were quantified by Bradford assay and an equal amount of each protein (40 μg) was separated by SDS-PAGE and then electrotransferred to a nitrocellulose membrane. The detection of different proteins was carried out using the alkaline phosphatase colorimetric or electrochemiluminescence (ECL) labeling system. Optical density of the bands was analyzed with Quantity One Imaging Software (Bio-Rad Laboratories, Hercules, CA, USA). The correct protein loading was verified by means of both Ponceau red staining and immunoblotting for the housekeeping protein γ-tubulin. The results of the densitometric analysis are reported as the ratio of the intensity of protein bands normalized to γ-tubulin versus the band intensity of untreated samples. The results shown are representative of three independent experiments with similar results.

### 2.10. Gene Silencing

A pool of three target-specific small interfering RNAs (siRNAs) against Beclin-1 (siBeclin) (sc-29797) and a scrambled siRNA (siScr), as a negative non-silencing control, were purchased from Santa Cruz Biotechnology (Santa Cruz, CA, USA). Cells (1 × 10^5^) were seeded in 6-well plates and cultured in antibiotic-free RPMI 1640 medium supplemented with 2.0 mM glutamine, until approximately 50% confluence was reached [15]. Then, cells were transfected with 40 nM siBeclin in the presence of 4 μL Lipofectamine (Invitrogen Life Technologies, Monza, Italy) in a final volume of 1 mL serum-free medium. The reaction was stopped after 6 h by replacing the medium. After 24 h, silenced cells were treated with the litchi extracts for an additional 24 h.

### 2.11. Reversed Phase HPLC, MS and MS/MS Experiments

Water and acetonitrile were of HPLC/MS grade. Formic acid was of analytical quality. Samples for HPLC were prepared dissolving 1 mg of lyophilized fruit fractions with MeOH (1 mL). The HPLC system was an Agilent 1260 Infinity. A reversed-phase Phenomenex Luna C18(2) column (150 mm × 4.6 mm, particle size 3 µm) with a Phenomenex C18 security guard column (4 × 3 mm) was used. The flow-rate was 0.5 mL/min and the column temperature was set to 30 °C. The eluents were formic acid–water (0.1:99.9, v/v) (phase A) and formic acid–acetonitrile (0.1:99.9, v/v) (phase B). The following gradient was employed: 0–5 min, 5% B isocratic; 5–15 min, linear gradient from 5% to 15% B; 15–20 min, 15% B isocratic; 20–25 min, linear gradient from 15% to 30% B; 25–35 min, 30% B isocratic; 35–45 min, washing and reconditioning of the column to 5% B. Injection volume was 25 µL. The eluate was monitored through Mass Total Ion Count (MS TIC). Mass spectra were obtained on an Agilent 6540 UHD accurate-mass Quadrupole- Time of flight (Q-TOF) spectrometer equipped with a Dual AJS Electrospray Inonization (ESI) source working in negative mode. Nitrogen N_2_ was employed as desolvation gas at 300 °C and a flow rate of 8 L/min. The nebulizer was set to 45 psig. The sheath gas temperature was set at 400 °C and a flow of 12 L/min. A potential of 2.6 kV was used on the capillary for negative ion mode. The fragmentor was set to 75 V. MS spectra were recorded in the 150–1000 *m*/*z* range.

### 2.12. Statistical Analysis

The analysis was performed using the statistical software package GraphPad Prism4 (San Diego, CA, USA). The data obtained was analyzed with Student’s *t* test or one-way analysis of variance (ANOVA). The data is expressed as means ± SD. Differences between groups were considered to be significant at a *p* value < 0.05.

## 3. Results

### 3.1. Quantification of Polyphenol Content in Litchi Extracts

In the first phase of this study, we evaluated the amount of polyphenols in Sicilian litchi fruit extracts by means of a colorimetric assay, which used gallic acid as a standard. The results indicated that the amounts of polyphenols in hydro-alcoholic litchi extracts are equal to 113.3 mg/g of dry weight in litchi exocarp extract, 93.61 mg/g in endocarp and 75.83 mg/g in mesocarp. The concentrations of Litchi extracts used in the experiments were then expressed as µg gallic acid equivalents (GAE)/mL of culture medium.

### 3.2. Litchi Extracts Induce Cell Viability Reduction and Growth Inhibition in Colon Cancer Cells

**Figure 1:**
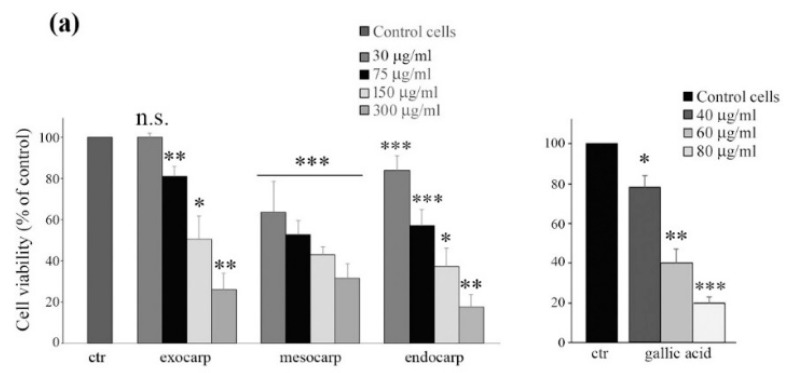

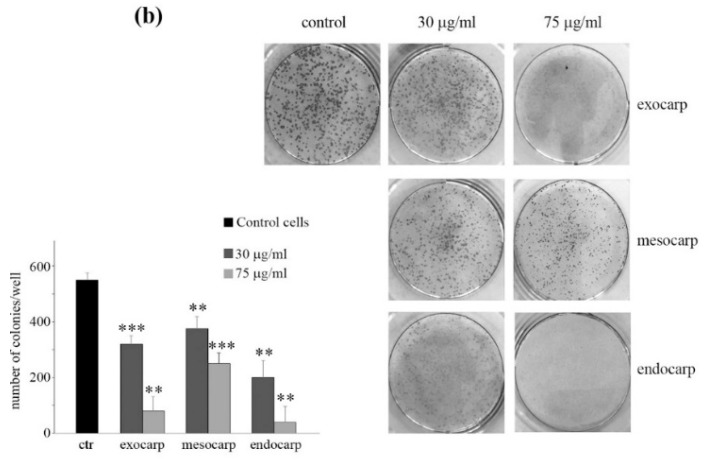
Effects of litchi extracts on HT29 cell viability and clonogenic ability. (**a**) Cells (7 × 10^3^) were exposed to different doses of litchi extracts (left panel) or gallic acid (right panel) and the incubation was protracted for 48 h. Cell viability was then assessed by the colorimetric 3-(4,5-dimethyl-thiazol-2-yl)-2,5-diphenyltetrazolium bromide (MTT) assay as reported in the Materials and Methods. (**b**) Single cell suspensions (300 cells) were seeded in 6-well plates and were treated with two different doses of litchi extracts after 48 h. A clonogenic assay was carried out after eight days and the number of colonies was estimated as reported in the Materials and Methods. The results reported in the histograms are representative of three separate experiments. (*) *p* < 0.05; (**) *p* < 0.01; (***) *p* < 0.001 compared with control (ctr). n.s. not significant.

The effects of different doses of litchi extracts on colon cancer HT29 cell viability were evaluated by MTT assay as reported in Figure 1a. All fractions of litchi decreased cell viability in a dose-dependent manner, reaching the maximum cytotoxic action at 48 h of treatment with 300 µg GAE/mL exocarp (−74%), mesocarp (−69%) and endocarp (−83%). Similar effects were obtained after treatment with increasing doses of gallic acid (Figure 1a, right panel).

In addition, a clonogenic assay was performed to further validate the inhibiting effect of litchi extracts on HT29 cells. As shown in Figure 1b, cells treated for eight days with low doses of extracts showed a significant dose-dependent inhibition of colony formation compared to the untreated controls. According to MTT analysis, the maximum effect was observed with litchi endocarp extract (about 95% inhibition of colony formation with 75 µg GAE/mL, *p* < 0.01).

### 3.3. Growth Inhibition of Colon Cancer Cells is Due to Cell Cycle Arrest and Apoptotic Cell Death

Based on the results reported in Figure 1a, all the subsequent experiments were carried out in HT29 cells using 150 µg GAE/mL for each fraction of the fruit (the dose that induced about 40–50% reduction in cell viability after 48 h treatment).

**Figure 2:**
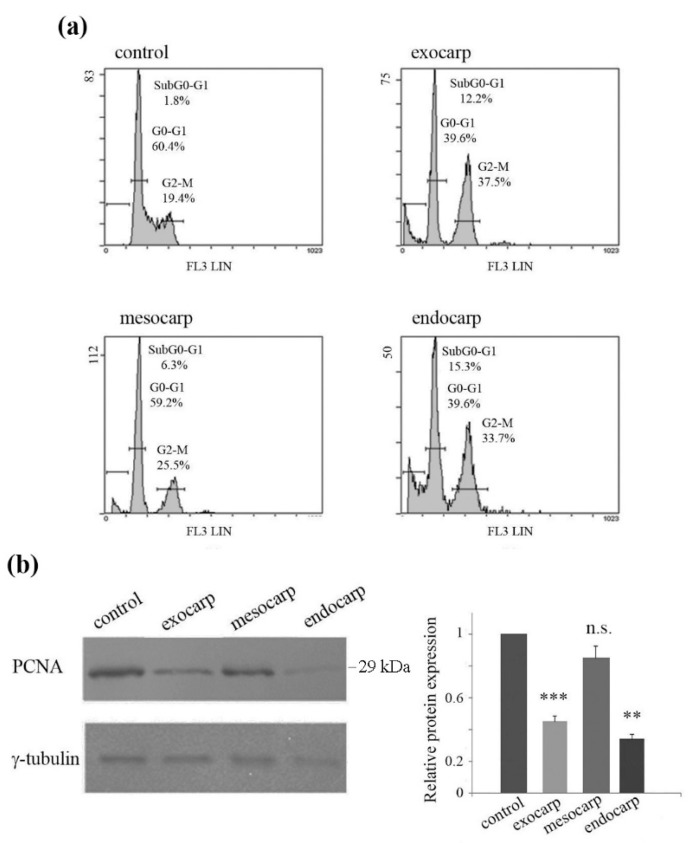
Effects of litchi extracts on cell cycle distribution and nuclear proliferating cell antigen (PCNA) level. (**a**) HT29 cells were exposed to 150 µg/mL gallic acid equivalents (GAE) litchi exocarp, mesocarp or endocarp treatment for 48 h, then the DNA content was evaluated by flow cytometry after staining the cells with a hypotonic propidium iodide solution. The graphs show the percentage of cells, calculated by the Expo 32 software, in the different phases of the cell cycle as described in the Materials and Methods. (**b**) Western blotting of PCNA levels after treatment with the litchi extracts. The correct protein loading was ascertained by immunoblotting for γ-tubulin. Representative blots of three independent experiments and densitometric analysis are shown. (**) *p* < 0.01; (***) *p* < 0.001 compared with control; n.s. not significant.

To elucidate the underlying mechanism of the effects of litchi extracts, we analyzed the cell cycle distribution of HT29 cells. After 48 h of treatment with litchi extracts, the cells appeared to be arrested in the G2/M phase of the cell cycle, in particular in the presence of exocarp and endocarp extracts (37.5% and 33.7% respectively, vs. 19.4% in untreated cells) (Figure 2a). The anti-proliferative effects of the litchi portions in HT29 cells were also confirmed by the evidence that proliferating cell nuclear antigen (PCNA) significantly decreased in the presence of exocarp (*p* < 0.001) and endocarp (*p* < 0.01), and to a minor extent with mesocarp extract (Figure 2b). This finding was in accordance with the results obtained by MTT assay and cell cycle evaluation.

**Figure 3:**
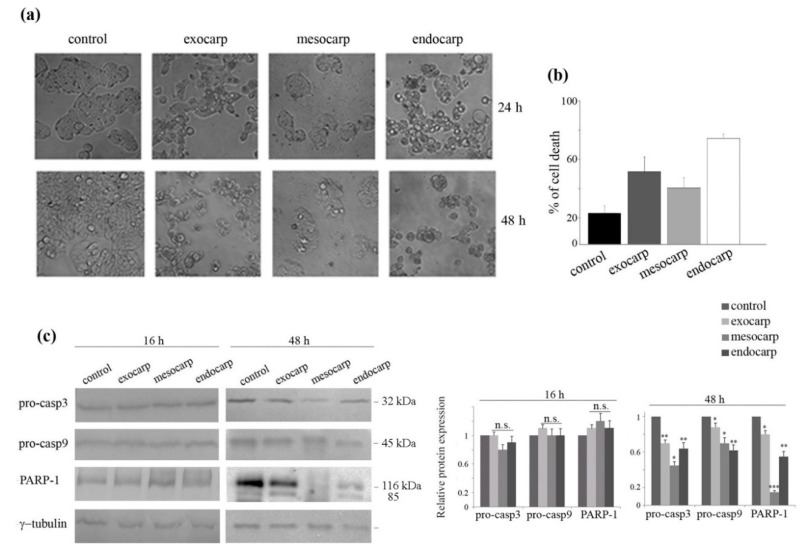
Apoptotic effects promoted by litchi fruit extracts in HT29 cells. (**a**) Morphological effects induced by litchi extracts in HT29 cells. The pictures were acquired after cell treatment with 150 µg/mL GAE litchi extracts at the indicated times by a Leica light microscope (200× magnification). (**b**) Detection of HT29 cell death. After 48 h of treatment, cells were incubated with an isotonic propidium iodide (PI) solution for 1 h and analyzed by flow cytometry using the Expo 32 software. The histograms indicate the percentage of PI-positive cells that reveal membrane permeabilization accounting for cell death. *p* value < 0.001. (**c**) Western blot analysis of apoptotic proteins evaluated after 16 h and 48 h of treatment. The correct protein loading was ascertained by immunoblotting for γ-tubulin. Representative blots of three independent experiments and densitometric analysis are shown. (*) *p* < 0.05; (**) *p* < 0.01; (***) *p* < 0.001; n.s. not significant.

The profile of cell cycle distribution also revealed the appearance of the subG0/G1 peak at 48 h treatment, indicating DNA fragmentation and induction of cell death (Figure 2a). The analysis of cell morphology confirmed the appearance of apoptotic features after prolonged treatment times, consisting of cell isolation, shrinkage and detachment from the substrate (Figure 3a, lower panel). Cell death induction was also highlighted by the exposure of the cells to isotonic propidium iodide solution (Figure 3b). As a further confirmation of apoptosis induction, a decrease in the levels of procaspase-3 and procaspase-9 was found together with the degradation of the specific caspase-3 substrate poly-ADP-ribose-polymerase-1 (PARP-1). These effects were observed after 48 h of treatment, but were not evident in the early time point (16 h), suggesting that the execution of apoptosis is a late event. (Figure 3c).

Preliminary results indicated that pre-treatment of the cells with the pan-caspase inhibitor carbobenzoxy-valyl-alanyl-aspartyl-[O-methyl]-fluoromethylketone) (z-VAD-fmk) increased the effects of litchi mesocarp and only partially reduced the effect of exocarp and endocarp.

### 3.4. Induction of Autophagic Flux by Litchi Exocarp and Endocarp

Visualization of cell morphology after a short treatment period (16–24 h) with litchi extracts indicated that the exocarp and endocarp provoke the appearance of big cytoplasmic vacuoles (Figure 3a, upper panel), which are not evident in the presence of litchi mesocarp. In light of this observation, we speculated that litchi exocarp and endocarp extracts, besides the induction of apoptosis, could also function to induce autophagy.

**Figure 4:**
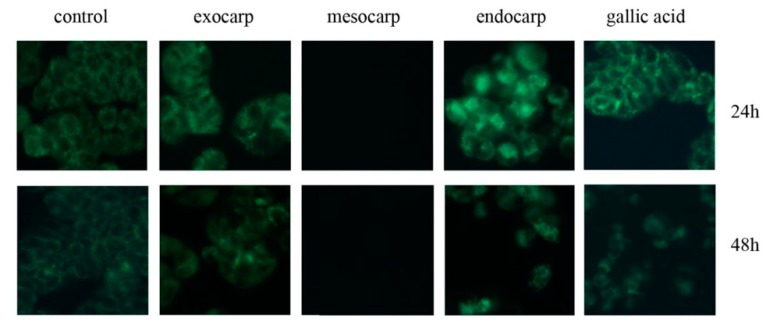
Litchi exocarp and endocarp extracts induce intracellular vacuolization. Cells (7 × 10^3^) were treated with 150 µg/mL GAE litchi extracts or 60 µg/mL gallic acid for the indicated times. Afterward, cells were incubated with 50 µM monodansylcadaverine (MDC) for 10 min, washed with phosphate-buffered saline (PBS) and visualized under a fluorescence microscope equipped with a 4′,6-diamidino-2-phenylindole (DAPI) filter at a magnification of 400×. Micrographs are representative of almost three fields from two independent experiments.

To verify this hypothesis, cells were stained with MDC, a fluorescent compound that labels autophagic vacuoles. Positive results with MDC staining consisted of the appearance of dot-like structures inside the cells. This was clearly evident at 24 h treatment with exocarp and endocarp extracts (Figure 4, upper panel). Also in this case, the maximum effect was evident in the presence of endocarp. On the other hand, autophagic features did not appear at all in cells treated with mesocarp extract. Interestingly, gallic acid also positively determined MDC staining. Prolonging the treatment time to 48 h resulted in the attenuation of autophagic vacuole staining, most likely due to increasingly progressive cell death (Figure 4, lower panel).

**Figure 5:**
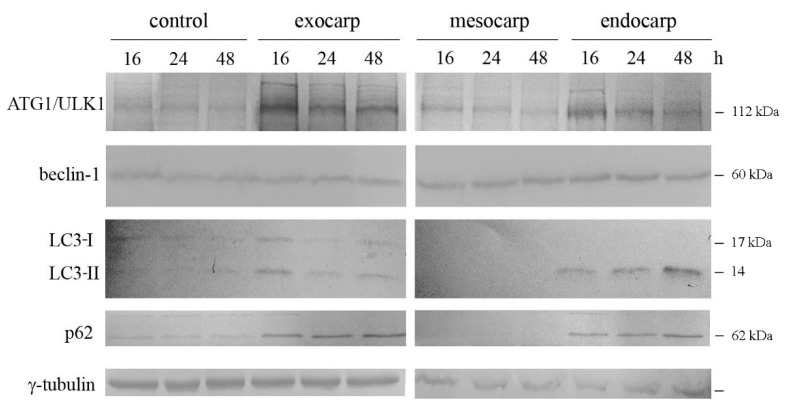

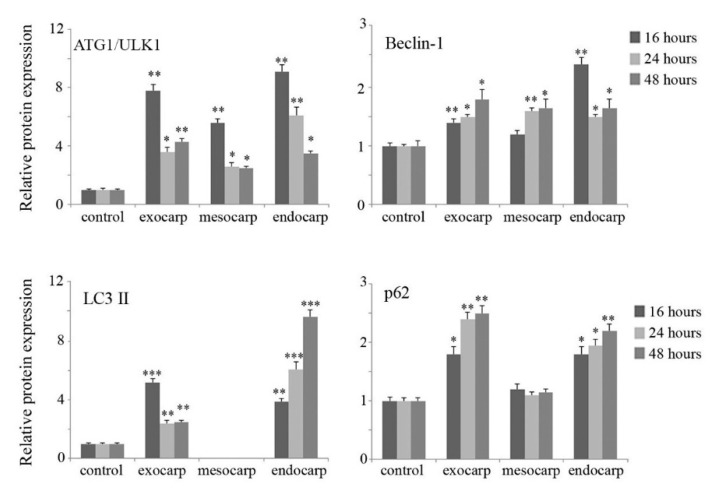
Time course evaluation of autophagic markers after treatment with litchi extracts. Cells were treated for the indicated times with 150 µg/mL GAE litchi exocarp, mesocarp or endocarp extracts. The cells were then harvested and protein extracts were prepared and subjected to western blotting analysis to determine the levels of autophagic markers. ATG1/ULK1 (autophagy related 1/Unc-51 like autophagy activating kinase), LC3 (microtubule associated protein 1 light chain 3). The correct protein loading was ascertained by immunoblotting for γ-tubulin. Representative blots of three independent experiments are shown. Densitometric analyses are present in supplementary data. (*) *p* < 0.05; (**) *p* < 0.01; (***) *p* < 0.001 compared with control.

To confirm the induction of autophagic flux and to follow its evolution, we performed a western blot time point evaluation of some key proteins. As shown in Figure 5, a remarkable increase in the kinase ATG1/ULK1 occurred when the cells were treated with exocarp and endocarp extracts. This effect appeared early at 16 h of treatment and slightly decreased at 24 h and 48 h. In addition, increases in the level of beclin-1, a protein involved in the first phase of autophagy, were observed particularly in cells treated with litchi endocarp extract.

The microtubule-associated protein 1 light chain 3 (LC3) is considered to be a marker of autophagosome presence [27]. This factor is activated by cleavage and lipidation of the cytosolic form LC3-I to LC3-II, and is then recruited to the membrane of the autophagosome. When the cells were treated with litchi exocarp, the induction of both LC3-I and LC3-II was observed at 16 h and tended to decrease after longer periods of time. With litchi endocarp, a marked progressive increase in the autophagic LC3-II form was observed, which reached the maximum at 48 h (Figure 5, *p* < 0.001).

The analysis of p62, a multifunctional protein involved in selective autophagy and considered as a marker to study autophagic flux [28], was shown to increase with both litchi exocarp and endocarp which was maintained at each time point (Figure 5).

According to the results obtained with the MDC assay, no significant effect was observed on autophagic protein levels when the cells were treated with litchi mesocarp.

### 3.5. Inhibiting Autophagy Promotes Cell Death

**Figure 6:**
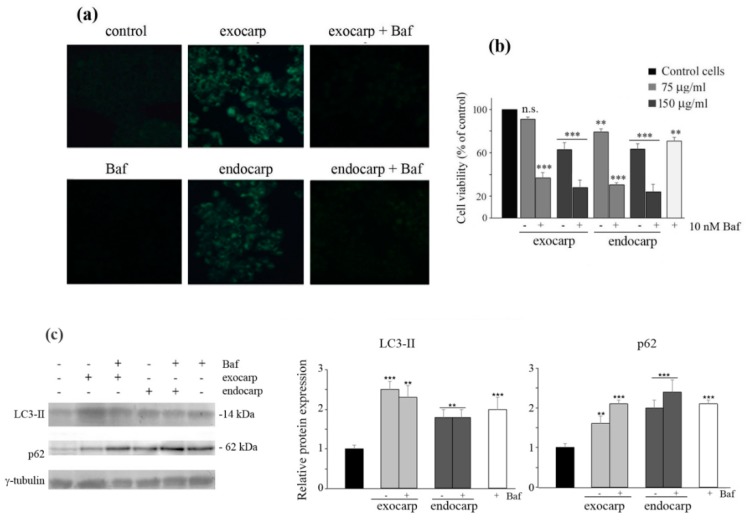
Bafilomycin A1 treatment increases cytotoxic effects of litchi extracts in HT29 cells. (**a**) The efficacy of the autophagy inhibitor bafilomycin A1 (Baf) (10 nM) in preventing autophagy was ascertained by MDC staining as reported in Figure 4. Micrographs are representative of three independent experiments (200× magnification). (**b**) Cells (7 × 10^3^) were treated with the indicated doses of litchi exocarp or endocarp extracts either in the presence or absence of bafilomycin A1 for 24 h. Cells were then subjected to an MTT assay as reported in the Materials and Methods. (**) *p* < 0.01; (***) *p* < 0.001 compared with control. (**c**) Western blotting of LC3-II and p62 after treatment of HT29 cells with the litchi extracts in the presence or absence of bafilomycin A1 for 24 h. The correct protein loading was ascertained by immunoblotting for γ-tubulin. Representative blots of three independent experiments and densitometric analysis are shown. (**) *p* < 0.01; (***) *p* < 0.001 compared with control.

To clarify the role of autophagy observed after exposure to exocarp and endocarp extracts, co-treatment of cells with bafilomycin A1, an autophagy inhibitor, was carried out. First, MDC staining confirmed that the inhibition of autophagy by bafilomycin A1 clearly reduced the number of fluorescent cells (Figure 6a). Then, an MTT assay showed that the cytotoxic effect of litchi extracts was considerably increased by the addition of bafilomycin A1 (Figure 6b), suggesting that autophagy has a pro-survival significance in these experimental conditions. Similar effects were observed when cells were pre-treated for 2 h in the presence of bafilomycin A1.

Then, we performed western blot analysis of LC3-II and p62 in the presence of bafilomycin A1. As expected, treatment with bafilomycin alone increased the levels of both proteins. Interestingly, bafilomycin A1 did not modify the effects induced by the extracts on LC3-II, while it further increased the levels of p62, thus confirming that after an early stimulation of autophagy, the extracts induced an arrest of the autophagic flux, leading to the accumulation of the proteins (Figure 6c).

**Figure 7:**
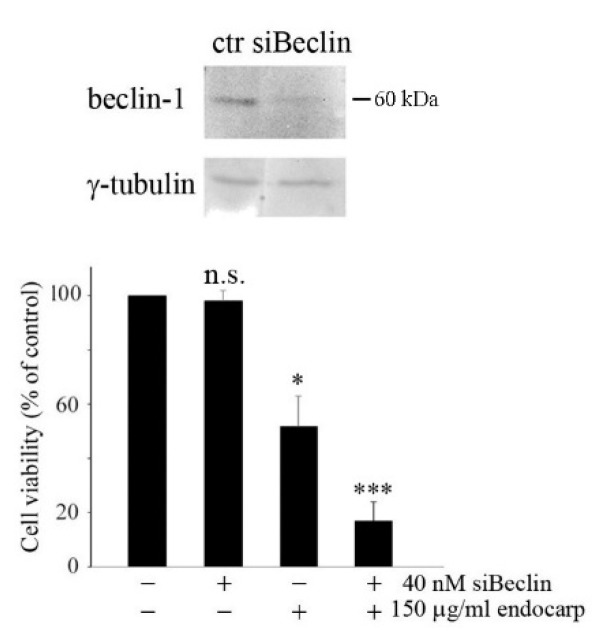
Beclin silencing counteracts the cytotoxic effects of litchi extracts. After silencing, siRNA against beclin-1 (siBeclin) and scramble control transfected cells were subjected to western blot analysis to verify beclin-1 silencing. An MTT assay was performed after treating transfected cells with litchi endocarp extracts. All histograms shown are representative of three independent experiments. (*) *p* < 0.05; (***) *p* < 0.001 compared with control.

To confirm the pro-survival significance of autophagy induced by litchi exocarp and endocarp, cells were transfected with a specific siRNA against beclin-1. After confirming by western blot analysis that beclin-1 silencing reduced the protein level (Figure 7), silenced cells were treated with litchi endocarp extract and cell viability was estimated by MTT assay. As shown in Figure 7, in silenced cells, the viability of treated HT29 cells was further reduced from 49% (*p* < 0.05) to 14% (*p* < 0.001).

### 3.6. Effects of Litchi Extracts on Other Colon Cancer Cells and Non Tumor Counterparts

**Figure 8:**
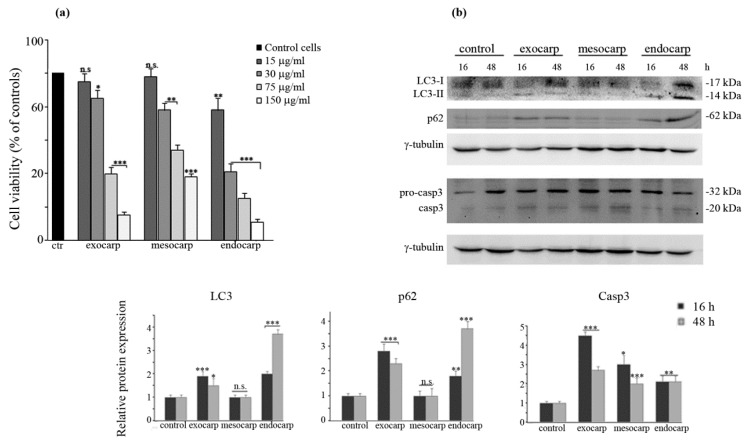
Effects of litchi fruit extracts on cell viability and autophagic and apoptotic markers in HCT116 cells. (**a**) Cells (7 × 10^3^) were exposed to different doses of litchi exocarp, mesocarp or endocarp extracts and the incubation was protracted for 48 h. Cell viability was then assessed by a colorimetric MTT assay as reported in the Materials and Methods. The results are representative of three independent experiments. (*) *p* < 0.05; (**) *p* < 0.01; (***) *p* < 0.001 compared with control. (**b**) Western blotting of LC3, p62 and caspase-3 after treatment of HCT116 cells with litchi extracts (75 µg/mL) for 16 h or 48 h. The correct protein loading was ascertained by immunoblotting for γ-tubulin. Representative blots of three independent experiments and densitometric analysis are shown. (*) *p* < 0.05; (**) *p* < 0.01; (***) *p* < 0.001 compared with control.

**Figure 9:**
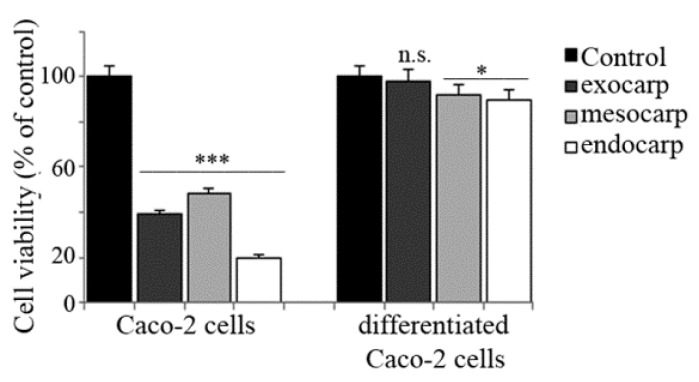
Comparative effects of litchi extracts on parental and differentiated Caco-2 cell viability. Caco-2 cells (3 × 10^5^ cells/cm^2^) were seeded on polyethylene terephthalate (PET) membrane inserts and allowed to differentiate for 21 days. Twenty-four hours before treatment, parental Caco-2 cells were also plated on PET membrane inserts. Then, the two cell lines were treated with 150 µg/mL of each litchi extract. The incubation was protracted for an additional 48 h and the cells were then subjected to an MTT assay as reported in the Materials and Methods. The results are representative of three independent experiments. (*) *p* < 0.05; (***) *p* < 0.001 compared with control.

In order to strengthen the anti-tumor potential and specificity of litchi extracts, in a final phase of our research, we extended our attention to HCT116 and Caco-2 colon cancer cells as well as a non-tumor model of human enterocytes obtained by differentiating Caco-2 cells accordingly to Natoli et al. [22]. The results of the MTT assay indicated that both HCT116 and parental Caco-2 cells were highly responsive to litchi extracts (Figure 8 and Figure 9) while differentiated Caco-2 cells were not sensitive at all (Figure 9).

Moreover, we evaluated the effects of litchi extracts on the levels of some autophagic and apoptotic markers in HCT116 cells. The results of western blotting analysis showed that, similarly to the results obtained in HT29 cells, both litchi exocarp and endocarp increased the autophagic LC3-II form, as well as p62 levels, in HCT116 cells. On the other hand, litchi mesocarp did not induce autophagic markers. Instead, all the litchi fractions decreased the pro-caspase-3 levels at 48 h, thus suggesting a late execution of apoptosis (Figure 8).

### 3.7. Contents of Polyphenols in Litchi Exocarp, Mesocarp and Endocarp Extracts and Their Individual Composition

**Figure 10:**
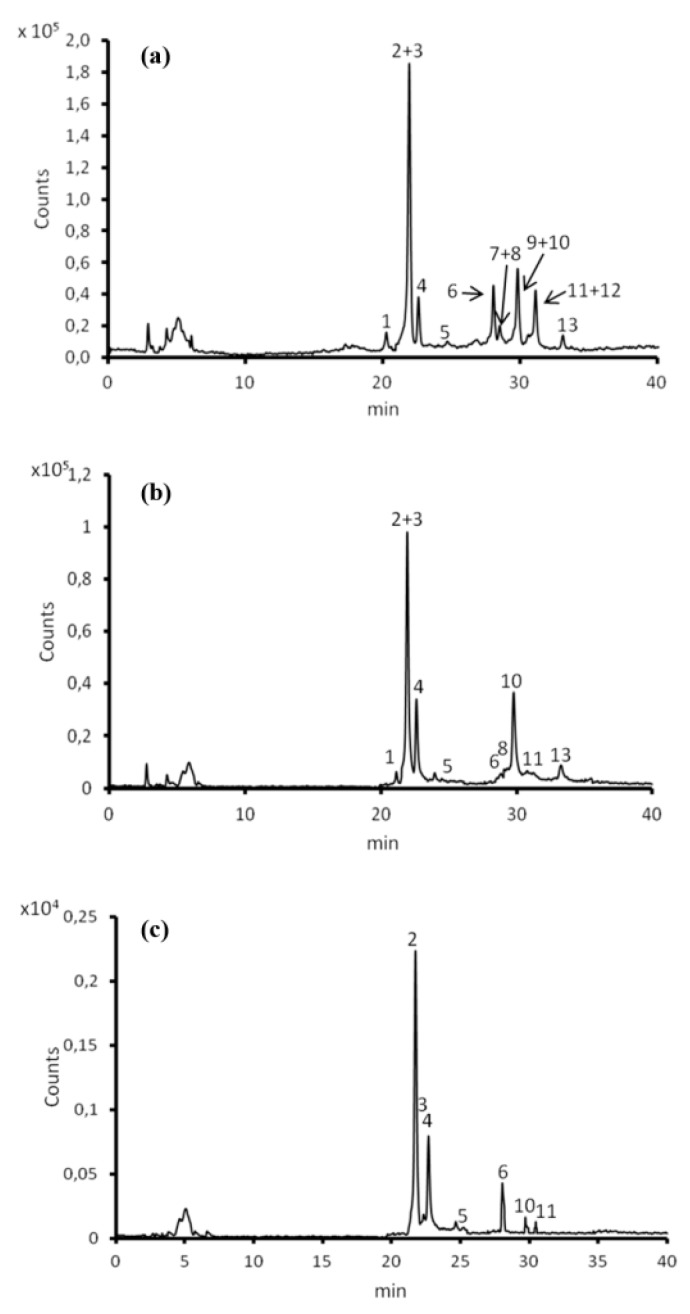
Representative High-Performance Liquid Chromatography–Electrospray Ionization-Quadrupole-Time-Of-Flight (HPLC/ESI/Q-TOF) trace of extracts: exocarp (**a**), endocarp (**b**) and mesocarp (**c**).

Finally, we characterized litchi extracts through HPLC/ESI/Q-TOF experiments [29]. The HPLC/MS analysis allowed the detection of 13 different compounds (Figure 10 and Table 1) which were differently represented in the three parts of litchi fruits from both a quantitative and a qualitative point of view.

Flavan-3-ols, small oligomers of epicatechin, are principal compounds (84.7%, 85.2% and 97.7% of the totality of the polyphenolic component in exocarp, mesocarp and endocarp, respectively). Epicatechin (**2**), its dimer procyanidin A2 (**10**) and its trimer pavetannin B2 (**4**) are the three main proanthocyanidins of litchi fruit parts, which was consistent with the results previously reported [29]. These three compounds represent almost all of the total phenols present (about 80% in the three fractions). The flavonol glycosides constitute 14.7% and 2.4% of the total polyphenolic component in the mesocarp and in the endocarp, but are represented exclusively by rutin (quercetin-3-*O*-rutinoside) (**6**). In the exocarp, the flavonol glycosides are 10.5% of the phenolic component and there are three different compounds (**6**, **7**, **9**).

Only one anthocyanin, cyanidin-3-rutinoside, was detected exclusively in the exocarp, such as previously reported [29]. The quali-quantitative anthocyanin profiles may reflect cultivar differences.

## 4. Discussion

This paper describes for the first time the effects of different fractions of Sicilian litchi fruit in human colon cancer cells. In particular, the results indicate that litchi extracts determine inhibition of HT29 cell proliferation in a dose-dependent manner, an effect that was due to cell cycle arrest in the G2/M phase and cell death induction. These results are in accordance with the observations of Hsu et al. [9] who have shown that litchi seed extracts induce cell cycle arrest and apoptosis in human colorectal carcinomas by decreasing the expression of cyclins, altering the Bcl-2 family member ratio towards pro-apoptosis and activating caspase-3. Our study extended the attention to the three main fractions of litchi fruit including the peel (exocarp), the pulp (mesocarp) and the seed (endocarp).

It is well known that all the fractions separated by litchi fruit contain bioactive compounds, including polyphenols, which display antioxidant properties and can exert anti-tumor effects [30,31,32]. Purified components of this class or specific mixtures reduce cancer cell proliferation and induce apoptosis [31], suggesting that polyphenol-rich litchi extracts could have an effect on cancer prevention and treatment. In fact, nowadays, the growing incidence of cancer in developed countries, as well as its tight correlation to cancer-related deaths, has pushed scientists to explore new possible therapeutic alternatives to deal with this challenge. Under this view, the anti-neoplastic action of natural compounds bearing multiple polyphenol rings has been strongly highlighted. Polyphenols are endowed with a broad spectrum of structural variations in the carbon backbone chains that make them responsible for various health benefits, ranging from anti-cancer to anti-inflammatory properties. These aspects, in accordance with their selective toxicity in tumor systems, have also suggested polyphenol use as anti-cancer or chemopreventive drugs to overcome the side effects and resistance observed with the mostly-used chemotherapeutics.

In this study, our data provided evidence that all the three litchi coarse extracts determined anti-proliferative effects in HT29 cells and altered the membrane permeability to propidium iodide, thereby indicating cell death. We also provided evidence that the cells underwent cell death through a G2/M block of the cell cycle, an effect particularly evident with litchi exocarp and endocarp extracts and in accordance with the remarkable decrease in the level of PCNA.

Interestingly, litchi exocarp and endocarp clearly triggered an autophagic response, as shown by MDC staining and the detection of autophagic markers. This paper represents the first evidence that litchi extracts are capable of inducing autophagy. In particular, in the first phase of treatment (16–24 h) we observed that litchi exocarp and endocarp determine the appearance of autophagosomes, as evidenced by the presence of dot-like structures distributed only in the cytosol of MDC-positive cells. This effect was accompanied by significant variations in the levels of the main autophagic markers, such as ATG1/ULK1 kinase and its targets beclin-1, LC3 and p62.

In regards to the autophagic LC3-II active form, a remarkable increase was observed with litchi exocarp extract at 16 h of treatment together with the LC3-I precursor, which also increased compared to the untreated control. Thus, we hypothesized that litchi exocarp can induce LC3 expression and, at the same time, promote its cleavage to pro-autophagic LC3-II. With litchi endocarp, only the active LC3-II form appeared and further accumulated until reaching the maximum at 48 h. Considering that autophagic effects were more evident in cells treated with the endocarp, it is possible to assume that complete conversion of LC3-I in the active LC3-II form is responsible for the greater effect observed after endocarp treatment.

We can hypothesize that autophagic flux activation is triggered by polyphenols contained in litchi exocarp and endocarp extracts. In accordance to this hypothesis, Pietrocola et al. [33] have demonstrated that polyphenols, including gallic acid, determine pro-autophagic effects in human osteosarcoma cells and reduce the acetylation of cytoplasmic proteins. In this paper, we show that treatment of HT29 cells with gallic acid mimicked the effects induced by litchi extracts on cell viability and promoted an autophagic response similar to those observed with exocarp and endocarp treatment.

The evidence that the induction of autophagy is a precocious event that precedes the appearance of apoptotic signs suggested that it could be a response of the cell to counteract the toxic effect induced by litchi treatment. The inhibition of the process using bafilomycin A1 in co-treatment with the extracts, or beclin-1 silencing, revealed a significant increase in the cytotoxic effect. Moreover, increased levels of both LC3-II and p62 under the inhibitory effect of bafilomycin A1 confirmed that blocking the autophagic process leads to the accumulation of these proteins. For such reasons, we conclude that the trigger of the autophagic flux was in correlation with a defensive mechanism activated by the cells in the initial phase of treatment. However, the process was not completed, as indicated by the levels of p62, a factor that functions as a selective autophagy receptor for degradation of ubiquitinated substrates [34,35]. p62 appeared in HT29 cells at 16 h treatment with the litchi exocarp and endocarp extracts and increased until 48 h. These results suggest that autophagy is initiated but not completed, most likely because the process switches to apoptosis in the second phase of treatment.

Apoptosis is the other possible event that accounts for the cytotoxic effects of litchi extracts in HT29 cells. In the current study, we showed that apoptotic effects clearly appeared following autophagy in the presence of exocarp and endocarp extracts and directly, without the induction of autophagy, with mesocarp extracts. Pro-apoptotic effects were clearly evident at 48 h treatment when caspase-3 and caspase-9 activation occurred, together with the degradation of PARP-1. However, considering our preliminary results in the presence of z-VAD-fmk, it is possible that other types of programmed cell death, different from apoptosis, take place. This hypothesis will be considered in a new study.

Since we carried out the experiments with equal amounts of GAE equivalent of the three litchi fruit fractions, it is possible to speculate that the different response obtained with the mesocarp extract is related to a more heterogeneous composition of this fraction.

Moreover, the characterization of polyphenolic components of the extracts evidenced that specific components (procyanidin B2, procyanidin B4, procyanidin A isomer) are present in exocarp and endocarp that are not present in mesocarp and another procyanidin (procyanidin A2) is at a higher concentration. Interestingly, evidence has been provided in the literature that most of these components exert anti-tumor and pro-apoptotic effects [10,31]. Studies are in progress to isolate these components and evaluate their specific effects on apoptosis and autophagy in HT29 cells.

Taken together, the results presented in this paper demonstrated that Sicilian litchi fruit extracts induce cell death in human colon cancer HT29 cells. This event was preceded by autophagy with litchi exocarp and endocarp, and directly proceeded toward apoptosis with litchi mesocarp.

The anti-tumor potential of litchi extracts in colon cancer was corroborated in this paper using other colon carcinoma lines (HCT116 and Caco-2 cells) that were particularly responsive (even more than HT29 cells) to all the three extracts. In addition, the observation that the non-tumor counterpart (differentiated Caco-2 cells) was instead not responsive, sustains the specificity of the components present in the litchi extracts toward tumor cells. Studies are ongoing to characterize the biochemical mechanism underlying such differences.

## Figures and Tables

**Table 1:** Profiles of polyphenols in different litchi parts.

Compound	RT * (min)	ESI ** (M − H)-(m/z)	Compound	Area %			
				Exo	Endo	Meso	
**1**	20.24	577.1352 (M − H)^−^	Procyanidin B2	4.4	1.9	-	Flavan-3-ol
**2**	21.92	289.0716 (M − H)^−^	Epicatechin	45.4	40.1	55.2	Flavan-3-ol
**3**	22.05	863.1824 (M − H)^−^	Epicatechin trimer	1.0	1.7	0.6	Flavan-3-ol
**4**	22.58	863.1828 (M − H)^−^	Pavetannin B2	13.5	19.1	24.5	Flavan-3-ol
**5**	24.68	863.1832 (M − H)^−^	Epicatechin trimer	0.7	1.3	0.9	Flavan-3-ol
**6**	28.05	609.1463 (M − H)^−^	Rutin (quercetin-3-O-rutinoside)	7.2	2.4	14.7	Flavonol glycoside
**7**	28.46	593.1515 (M − H)^−^	Nicotiflorin (Kaempherol-3-rutinoside)	1.9	-	-	Flavonol glycoside
**8**	28.48	577.1350 (M − H)^−^	Procyanidin B4	0.9	1.2	-	Flavan-3-ol
**9**	29.56	463.0893 (M − H)^−^	Quercetin-glucoside	1.4	-	-	Flavonol glycoside
**10**	29.83	575.1191 (M − H)^−^	Procyanidin A2	16.4	25.1	2.7	Flavan-3-ol
**11**	30.53	863.1820 (M − H)^−^	Epicatechin trimer	1.1	1.3	1.3	Flavan-3-ol
**12**	31.13	593.1505 (M − H)^−^ 629.1276 (M + Cl − H)^-^	Antirhinin (Cyanidin-3-rutinoside)	4.8	-	-	Anthocyanin
**13**	33.12	575.1188 (M − H)^-^	Procyanidin A isomer	1.3	6.0	-	Flavan-3-ol

* RT: retention time; ** ESI: electrospray ionization.
